# Teleexercise for geriatric patients with failed back surgery syndrome

**DOI:** 10.3389/fpubh.2023.1140506

**Published:** 2023-03-23

**Authors:** Reza Alizadeh, Albert Thomas Anastasio, Ardalan Shariat, Mikhail Bethell, Gholamreza Hassanzadeh

**Affiliations:** ^1^Department of Anesthesiology and Pain, AJA University of Medical Sciences, Tehran, Iran; ^2^Department of Orthopaedic Surgery, Duke University, Durham, NC, United States; ^3^Department of Digital Health, School of Medicine, Tehran University of Medical Sciences, Tehran, Iran; ^4^Department of Anatomy, School of Medicine, Tehran University of Medical Sciences, Tehran, Iran; ^5^Department of Neuroscience and Addiction Studies, School of Advanced Technologies in Medicine, Tehran University of Medical Sciences, Tehran, Iran

**Keywords:** geriatric, failed back surgery syndrome, teleexercise, low back pain, occupational therapy

## Abstract

**Introduction:**

Failed back surgery syndrome (FBSS) is defined as back pain which either persists after attempted surgical intervention or originates after a spine surgery. There is a high risk of perioperative morbidity and a high likelihood of extensive revision surgery in geriatric patients with FBSS or post-laminectomy foraminal stenosis.

**Methods:**

There is a need for less invasive methodologies for the treatment of FBSS, such as patient-tailored exercise training, with attention to the cost and special needs of the geriatric patients with FBSS. This commentary will provide some background regarding teleexercise (utilizing an internet-based platform for the provision of exercise-related care) for FBSS and will propose three exercises which are easy to administer over online-based platforms and can be the subject of future investigation.

**Results:**

Given the documented benefits of regular rehabilitative exercises for patients with FBSS, the high cost of face-to-face services, and the need for infection mitigation in the wake of the COVID-19 Pandemic, teleexercise may be a practical and cost-beneficial method of exercise delivery, especially for geriatric patients with limitations in mobility and access to care. It should be noted that, prescription of these exercises should be done after face-to-face evaluation by the physician and careful evaluation for any “red flag” symptoms.

**Conclusion:**

In this commentary, we will suggest three practical exercise training methodologies and discuss the benefits of teleexercise for geriatric patients with FBSS. Future research should aim to assess the efficacy of these exercises, especially when administered through telehealth platforms.

## 1. Introduction

Lower back pain is one of the leading causes of disability worldwide resulting in widespread social and economic implications (1). Failed back surgery syndrome (FBSS) or post laminectomy syndrome (PLS) describes the condition of persistent pain following spine surgery, such as laminectomy, discectomy, or fusion-related procedures. FBSS is extremely common, with 74.6% of patients undergoing discectomy for lumbar disc herniation reporting residual lower back pain and 12% requiring an additional procedure (2). The potential etiologies of FBSS are complex and multifactorial, but residual lumbar foraminal stenosis is thought to contribute (3, 4). Symptoms related to FBSS can vary substantially across patients, but generally consist of pain and functional limitation (5). Geriatric populations have an increased risk for degenerative spinal disease leading to FBSS and foraminal stenosis. Moreover, higher rates of perioperative complications have been identified in geriatric patient populations undergoing spinal procedures (6, 7). Although recent investigations have explored endoscopic procedures for FBSS (3, 8, 9), noninvasive techniques have become a focal point of interest in the wake of the COVID-19 Pandemic.

Repeat spinal surgery as a treatment option does not guarantee a successful outcome: only 30, 15, and 5% of patients experience a good outcome after their second, third, and fourth surgery, respectively, (10). Therefore, due to the uncertainty of a good outcome in a repeat procedure, noninvasive techniques are favored when appropriate for management of FBSS. A systematic review found spinal cord stimulation to be an effective conservative treatment in decreasing disability and pain scores (11). Likewise, Lee et al. investigated the use of non-invasive painless signaling therapy with FBSS patients and found a decrease in cerebral pain perception (12). Wippert et al. did a randomized trials and found strong evidence supporting use of multidisciplinary rehabilitation exercises as an effective way to restore spinal function (13). Other non-pharmacological interventions such as hydrotherapy and guided strength training/stretching require specific facilities or in-person instruction. In addition, numerous reports have shown that multimodal exercises with cognitive behavioral therapy and intensive interdisciplinary pain rehabilitation can be utilized to improve disability in patients with FBSS (14, 15). In an effort to encourage the use of teleexercise for geriatric patients with FBSS, this commentary will (1) discuss the use of exercise for FBSS in geriatric patients and (2) propose 3 exercises for the treatment of FBSS which can be readily administered through online-based platforms.

## 2. Exercise for FBSS in geriatric patients

Regular exercise is imperative for geriatric individuals to maintain quality of life and functional independence (16). Aside from more obvious benefits such as improved muscle strength, flexibility, and cardiovascular health, exercise has positive effects on mood and can help mitigate psychological distress (17). In geriatric patients with FBSS, performing regular exercise may be especially difficult due to excess pain with movement, which may result in further deconditioning and worsening of their physical condition (18). Thus, managing the physical pain symptomology during exercise is critical to encourage regular patterns of exercise in patients with FBSS (19). With this in mind, several exercise protocols consisting of both passive and active movements have been developed for patients with FBSS. In passive forms of exercise, the patient does not have an active role and the process is carried out by a therapist, often using various devices (20). In contrast, active forms of exercise refer to those where a patient is engaged in carrying out a movement. Active exercise can be done either alone or with the aid of an assistant (21).

Active exercise therapy specifically has an important role in both pre- and postoperative spine care. Regular engagement with exercise protocols has been shown to treat back pain and to improve postoperative recovery after multiple spinal procedures (22). Thus, exercise is a cornerstone to the management of FBSS. Several studies have shown that exercise is a safe and effective treatment for this condition (23). Despite promising results, there is a need for more practical, evidence-based guidelines for exercise therapy which may be optimally employed in FBSS.

## 3. Teleexercise for FBSS in geriatric patients

In response to lockdowns from the COVID-19 Pandemic, a large number of medical consultations continue to occur through the use of online platforms (24). Geriatric patients may benefit particularly from health care delivery through online platforms, precluding risk of acquiring communicable diseases, and reducing transportation and access issues (25). While many exercise therapies have been proposed for FBSS (26), provision of guided exercise therapy through teleexercise has not been extensively studied (27). Moreover, geriatric populations who were not raised with the widespread use of internet communication platforms may struggle to adapt to these communication modalities.

There are several reports existing regarding the benefits of teleexercise for geriatric patients. Thus,with attention to the importance of core training in geriatric patients with FBSS (27), we propose 3 simple and practical exercises which are commonly used during in-person or online visits with patients who have FBSS and are referred to the Pain Clinic of Khatam Hospital (Tehran, Iran). These exercises were created with attention to Proprioceptive Neuromuscular Facilitation (PNF) (28) and follow basic principles for feasibility in various settings (29). Before participating in these exercises, careful evaluation from a physician should occur and one or two sessions should be performed in-person, under the supervision of a trained professional. Safety concerns should be explained to the patient and their assistant. At our institution, patients are interviewed and then screened and evaluated for suitability to an online-based treatment platform in a careful manner by a multidisciplinary team including a pain specialist, an occupational therapist, and an orthopedic surgeon.

Patients are initially screened for symptoms of FBSS including pain in the back, neck, or legs, or radiating pain. Moreover, patients are screened for “red flag” symptoms including excess mechanical pain which may indicate pseudarthrosis or non-union or accompanying fever and elevation in inflammatory markers which may indicate infected spinal hardware. If deemed appropriate for exercise therapy, patients are screened for appropriateness for video consultation. This screening includes questioning whether patients have appropriate technological knowledge to utilize video communication platforms and if they are comfortable with receiving therapy through these modalities. Once these screenings have been completed, a face-to-face visit occurs, ideally with the patient’s caretaker or assistant present, who may then later help the geriatric patient with FBSS at home. An educational video and poster are then sent to the patient electronically and their assistant can help with viewing. After this, teleexercise sessions begin. As other authors have noted, therapist participation during video therapy sessions allows for appropriate intervention and feedback to ensure that the patient is performing exercises in a safe and effective fashion (30). Online based sessions can then continue for as long as the patient requires active therapy engagement, with a focus on gradually transitioning the patient to performing the exercises on their own or with the aid of their at-home assistant.

Each exercise should be done 3 times a day, for 20 sets with a set duration of 10 s (for each leg/hand). While presence of an assistant is required for these exercises, these routines can be demonstrated through an online consultation, and then can be administered by a local caretaker.

### 3.1. Exercise 1: Hip abduction

**Figure 1:**
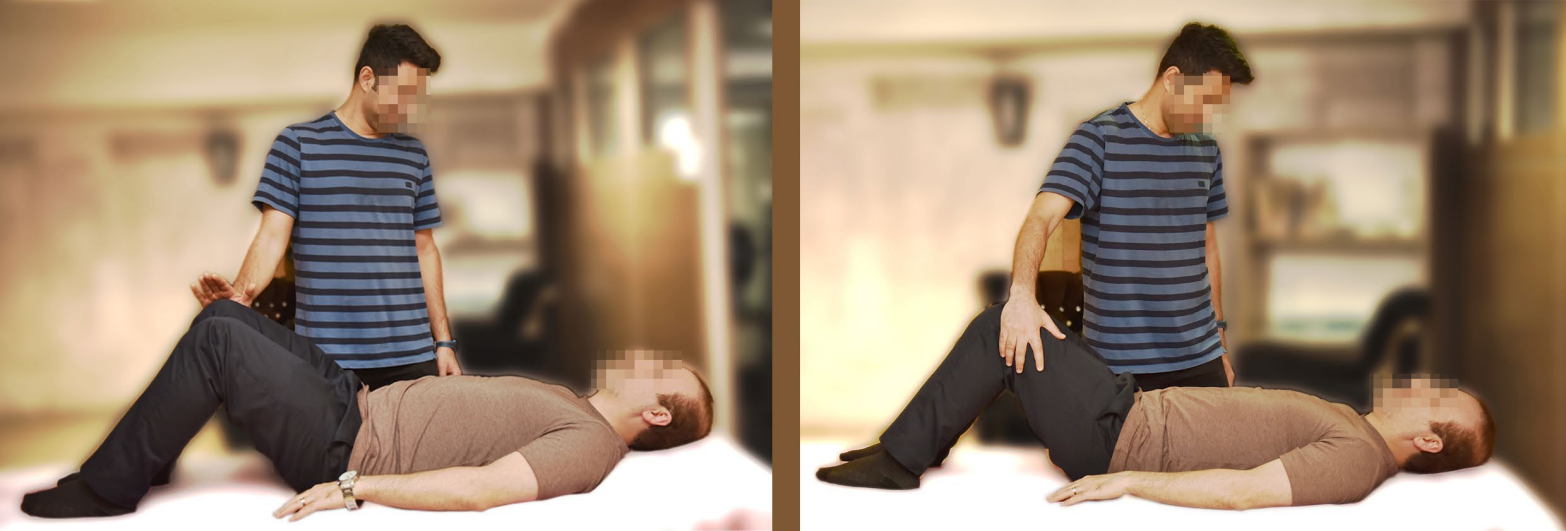
Hip abduction exercise.

The patient lays down and the assistant provides a force against the leg medially as demonstrated in the image. The patient attempts to counteract this force by pushing against the hand of the assistant. This exercise is repeated for both legs (Figure 1).

### 3.2. Exercise 2: Shoulder abduction

**Figure 2:**
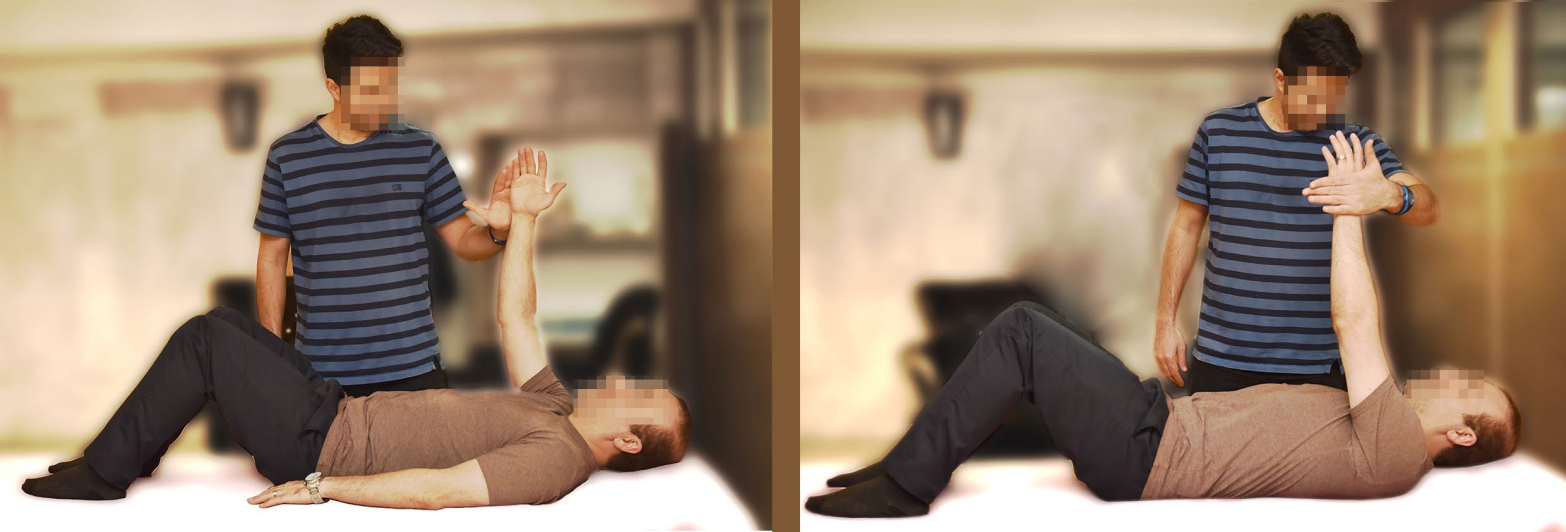
Shoulder abduction exercise.

The patient lifts one of their hands in a supine position while the assistant provides a force toward the patient’s midline as demonstrated in the image. The patient attempts to counteract that force by pushing against the assistant’s hand. This exercise is repeated for both hands (Figure 2).

### 3.3. Exercise 3: Hip and shoulder abduction exercise

**Figure 3:**
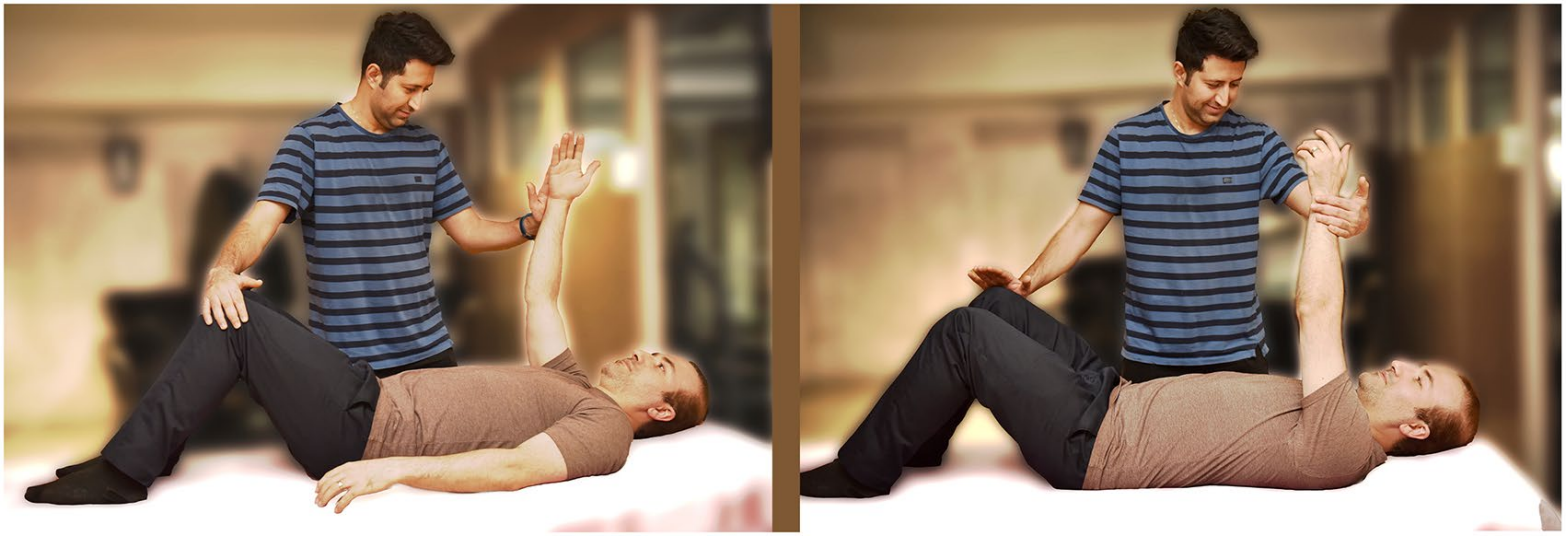
Hip and shoulder abduction exercise.

In the last exercise, the patient lifts their hand and contralateral leg in a cross position as demonstrated in the image. The assistant then provides a force against the patient’s hand and legs. This exercise is then repeated for the opposite side as well (Figure 3).

## 4. Research gaps and perspectives

The exercise suggestions in this paper should be considered as potential options for patients with FBSS. Randomized controlled trials are required to specifically investigate these techniques and to compare these movements to other exercise therapy regimens, especially with regards to suitability of care through teleexercise. In the wake of the COVID 19 Pandemic, consultation through online platforms is here to stay, and the provision of rehabilitative care must adapt to this changing landscape, with solid, evidence based protocols which have been validated through investigation of both in-person and online visits.

## Author contributions

AS and RA devised the project, the main conceptual ideas and proof outline. AT worked out almost all of the technical details. MB wrote the manuscript and GH finalized the paper. All authors contributed to the article and approved the submitted version.

## Conflict of interest

The authors declare that the research was conducted in the absence of any commercial or financial relationships that could be construed as a potential conflict of interest.

## Publisher’s note

All claims expressed in this article are solely those of the authors and do not necessarily represent those of their affiliated organizations, or those of the publisher, the editors and the reviewers. Any product that may be evaluated in this article, or claim that may be made by its manufacturer, is not guaranteed or endorsed by the publisher.
